# Percutaneous ultrasonographically guided liver punctures: an analysis of 1961 patients over a period of ten years

**DOI:** 10.1186/1471-230X-12-173

**Published:** 2012-12-05

**Authors:** Michael Mueller, Wolfgang Kratzer, Suemeyra Oeztuerk, Manfred Wilhelm, Richard Andrew Mason, Ren Mao, Mark Martin Haenle

**Affiliations:** 1 Department of Internal Medicine I, University Hospital Ulm, Albert-Einstein-Allee 23, 89081, Ulm, Germany; 2 Department of Mathematics, Natural Sciences and Economic Studies (Biostatistics), University of Applied Sciences Ulm, Albert-Einstein-Allee 55, 89081, Ulm, Germany; 3 Louis Stokes Cleveland Department of Veterans Affairs Medical Center, 10701 East Boulevard, Cleveland, Ohio, 44106, USA; 4 Department of Gastroenterology, The First Affiliated Hospital of Sun Yat-sen University, 58 Zhongshan II road, 510080, Guangzhou, P.R. China

**Keywords:** Liver, Biopsy, Ultrasonography, Complications, Risk factors

## Abstract

**Background:**

Ultrasonographically guided punctures of the liver represent a decisive tool in the diagnosis of many diseases of the liver. Objective of the study was to determine the extent to which the complication rate for ultrasonographically guided punctures of the liver is affected by less comprehensively studied risk factors.

**Methods:**

A total of 2,229 liver biopsies were performed in 1,961 patients (55.5% males; 44.5% females). We recorded actual complications and assessed the following risk factors: needle gauge, puncture technique, examiner experience, coagulation status, puncture target (focal lesion versus parenchyma), lesion size, patient sex and age.

**Results:**

he rate of complications stood at 1.2% (n = 27), of which 0.5% (n = 12) were major and 0.7% (n = 15) minor complications. A significant increase in complications involving bleeding was observed with larger-gauge needles compared with smaller-gauge needles and for cutting biopsy punctures compared with aspiration biopsies (Menghini technique). In the bivariate analysis complications were 2.7 times more frequent in procedures performed by experienced examiners compared with those with comparatively less experience. Lower values for Quick’s test and higher partial thromboplastin times were associated with a higher rate of bleeding. Neither the puncture target, lesion size or patient sex exerted any measurable influence on the puncture risk. Advanced patient age was associated with a higher rate of complications involving bleeding.

**Conclusions:**

Our study helps to establish the importance of potential and less comprehensively studied risk factors and may contribute to further reduction in complications rates in routine clinical practice.

## Background

Ultrasonographically guided percutaneous liver biopsies are an important diagnostic and therapeutic option in routine clinical practice [1-4]. They are employed in the work-up of patients with suspected diffuse liver diseases, for treatment monitoring and staging of hepatitis, and for diagnostic clarification of hepatic lesions [1]. With their limited invasivity, low rate of complications and brief post-procedure monitoring, these interventions can usually be performed on an ambulatory basis, which also contributes to their cost-efficiency [2,5,6]. The rate of minor complications is reported at 3.0-5.0%, with major complications occurring at a rate of 0.13-5.4% [7]. The major complication most commonly reported in the literature is bleeding [8]. The mortality rate is quite low, ranging from 0.0% to 0.4% [2,9,10]. However, many factors may influence the risk of complications and, to date, there is a paucity of quantitative data from studies with large patient collectives addressing certain risk factors. A recent published position paper by Rockey et al. [2] recognized by the American Association for the Study of Liver Diseases (AASLD) cites the evaluation of potential risk factors as an important topic for future studies. The present study addresses four of these: needle gauge, puncture technique, examiner experience and patient’s coagulation status. In addition, the following potential risk factors were assessed in the present study: target of biopsy (focal lesion, parenchyma), lesion size, and patient’s sex and age.

Objective of the present study was to establish the routine clinical relevance of these factors on the basis of prospectively collected data in a large patient collective in order to further minimize complication rates and assure the greatest possible safety for patients.

## Methods

### Nomenclature

A biopsy was defined as an intervention in which a single puncture target was approached through a single puncture channel using one or more puncture needles of the same or different diameter; one or more techniques could be used. The present report gives the external diameter of puncture needles in millimeters.

Major complications were defined as post-intervention events that were clinically relevant and required therapeutic intervention (e.g. blood transfusion, drainage etc.). Minor complications were events that were not clinically relevant and did not require specific therapeutic intervention.

### Patient selection

A total of 1,961 patients underwent 2,229 liver biopsies in our institution’s department of ultrasonography during the period of September 1999 through November 2009. In 31.5% (n = 702) of cases, liver biopsies were performed as out-patient procedures, while in 68.5% (n = 1527), biopsies were performed in inpatients.

The study was conducted in accordance with the principles of the Helsinki Declaration and Good Clinical Practice. It was approved by the ethics committee of the State Medical Council of Baden-Württemberg (74/12).

### Data collection

The following parameters were prospectively recorded: needle diameter, puncture technique, examiner’s experience (inexperienced: < 150 liver biopsies; experience: ≥ 150 liver biopsies), patient’s coagulation status (platelet count, Quick’s test, partial thromboplastin time [PTT]), target of puncture (focal lesion vs. parenchyma), size of focal lesions (size categories: < 2 cm, 2–5 cm, > 5 cm), patient’s sex, patient’s age (age groups: < 70 years; ≥ 70 years). For patients with complications, certain coagulation parameters were obtained retrospectively from the hospital’s medical record system.

### Puncture requirements

The following criteria had to be met before each puncture: clear indication with therapeutic consequence; adequate, current coagulation status (platelets ≥ 70,000/μl, Quick’s test value ≥ 70%, PTT < 50 seconds) no use of anticoagulants, and written consent signed by the patient one day prior to the procedure. In rare cases and after careful consideration of risks and benefits punctures were also performed in patients not meeting the required coagulation criteria. The greatest deviations from the routinely required coagulation parameters were observed in patients in critical condition; they are presented in Table 1.

**Table 1:** Complications and required interventions in 2,229 liver biopsies

**Description of complication**	**Indication**	**Final diagnose**
**Major complication (n=12)**		
Bleeding requiring transfusion (n=8)	Potentially malignant lesion (n=6)	Benigne liver tumor (n=3)
	Hepatopathy (n=1)	Hepatopathy (n=3)
	Hepatitis (n=1)	Metastases (n=2)
Vasovagal reaction (n=2)	potentially malignant lesion (n=1)	Metastases (n=1)
	Hepatitis (n=1)	Viral hepatitis (n=1)
Pneumothorax (n=1)	Autoimmune disorder (n=1)	No pathological findings (n=1)
Post-puncture anaphylactoid reaction (n=1)	Hepatopathy (n=1)	Hepatopathy (n=1)
**Minor complication (n=15)**		
Pain, vegetative symptoms (n=3)	Potentially malignant lesion (n=2)	Metastases (n=2)
	Hepatitis (n=1)	Hepatopathy (n=1)
Large hematoma of the liver (n=1)	potentially malignant lesion (n=1)	Metastases (n=1)
Reduction in hemoglobin concentration without evidence of bleeding (n=1)	Potentially malignant lesion (n=1)	Chonic hepatitis (n=1)
Significant perihepatic fluid (n=1)	Hepatopathy (n=1)	Non-diagnostic puncture (n=1)
mild bleeding (n=9)	Potentially malignant lesion (n=7)	Hepatopathy (n=2)
	Hepatitis (n=1)	Metastases (n=4)
	Hepatopathy (n=1)	No pathological findings (n=2)
		Poorly differentiated carcinoma (n=1)

### Execution

All punctures involving fine needle aspiration cytology (FNAC) or cutting biopsy (CB) were performed under ultrasonographic guidance (Philips HDI 3000/5000 Sono CT; C5-2 curved array transducers and biopsy guidance). The aspiration biopsy using the Menghini technique (ABM) was performed blind following initial ultrasonographic monitoring of the puncture track. The ABM was performed using a HEPAFIX Lok G 17 1.4-mm needle (B. Braun Melsungen AG, Melsungen, Germany). The intrahepatic phase of the needle insertion lasted 1–3 seconds.

For the FNAC, examiners used a puncture needle (Chiba, diameter: 0.7 mm, length: 220 mm; Bard Angiomed, Karlsruhe, Germany). A spinal needle (1.2 × 90 mm; Becton Dickinson S.M., Madrid, Spain) served as a leader canula. Following insertion of the leader canula, puncture material was obtained by the process of “needling”.

CB was performed in side notch technique using a fully automated biopsy pistol (Manan Medical Products, Inc., Wheeling, IL, USA) using Pro Mag 1.2 × 160 mm needles (Angiotech Pharmaceuticals, Inc., Vancouver, BC, Canada). The intrahepatic phase of both FNAC and CB required about 5–30 seconds. When during the same liver biopsy both a CB and FNAC were to be performed, the FNAC was always performed first.

### Patient monitoring after puncture

Following FNAC, patients were required to maintain bed rest for four hours (three hours after CB and ABM). In addition, they were requested to apply pressure to the puncture site with a sandbag for one hour (for 30 minutes after CB and ABM) and refrain from oral intake during the same period. Blood pressure and pulse readings were obtained every 60 minutes (every 30 minutes after CB and ABM) and recorded. Following final complete blood count, physical examination by the treating physician and final ultrasonographic examination following CB and ABM, patients were discharged. No sonographic examination was routinely performed following FNAC. Patients with acute pain or circulatory instability following discharge were advised to seek immediate medical attention in the Emergency Department.

### Statistical analysis

Statistical analysis was performed using the SAS statistical software version 9.2 (SAS Institute, Inc., Cary, NC, USA). Data was analyzed descriptively with respect to the complication rate and bleeding rate, absolute and relative frequency, mean, standard deviation, and maximum and minimum values. Potential risk factors were assessed for correlation with complication rate and bleeding rate using the χ^2^-test. If the absolute number of complications was too small, Fisher’s Exact Test was used. Subsequently, bivariate logistic regression was performed. Due to the small number of cases a multivariate analysis could not be performed. All statistical tests were two tailed. The significance level was set at α = 5% (p < 0.05).

## Results

A total of 2,229 liver biopsies were performed in 1,961 individual patients aged 18 to 96 years (mean 53.4 ± 16.1 years). 55.5% (n = 1,238) of liver biopsies were performed in male patients, 44.5% (n = 991) in female patients.

Hepatic lesions of unclear malignancy represent the most frequent indication for liver biopsy (50.5%). Punctures are also performed for therapy monitoring and staging of hepatitis (Table 2). The most frequent post-puncture diagnoses include diffuse liver diseases, such as chronic hepatitis or toxic liver damage, followed by viral hepatitis, carcinomas of hepatic origin and metastases (Table 2).

**Table 2:** Indication for liver biopsy and final diagnoses

**Indication**	**N (%)**
Potentially malignant lesion	1125 (50.5%)
Hepatitis	576 (25.8%)
Transaminase elevation	76 (3.4%)
Abscess/cyst	66 (3.0%)
Hepatopathy, hepatomegaly	161 (7.2)
Lymphoma	30 (1.4)
Metabolic disorders (Wilson’s disease, hemochromatosis, hemosiderosis)	30 (1.4)
Cirrhosis	22 (1.0)
Autoimmune disorder (autoimmune hepatitis, PBC, PSC)	87 (3.9)
Rejection reaction after liver transplantation	4 (0.2)
Jaundice of unclear origin	1 (0.04)
Thalassemia	4 (0.2)
Echinococcus infestation	3 (0.1)
NASH, ASH, steatosis	13 (0.6)
Cholestasis	10 (0.5)
Sarcoidosis	11 (0.5)
Bilioma	2 (0.1)
Hemangioma	1 (0.04)
Ascites	3 (0.1)
Still’s disease	1 (0.04)
Alpha-1 antitrypsin deficiency	2 (0.1)
Vasculitis	1 (0.04)
**Final diagnoses**	N (%)
Diffuse liver diseases	
*Viral hepatitis*	336 (15.1%)
*Autoimmune disorders*	43 (1.9%)
*Hepathopathy (NASH, ASH, steatosis, chronic hepatitis, drug-induced liver damage, nutritional-toxic damage, fibrosis, cirrhosis, liver necrosis, hepatosis, cholangitis, cholestasis, Budd-Chiari Syndrome)*	624 (28.0%)
*Metabolic disorders*	12 (0.5%)
*Rejection reaction following liver transplantation*	5 (0.2%)
*Sarcoidosis*	3 (0.1%)
Carcinomas of hepatic origin (HCC, CCC)	112 (5.0%)
Benign liver tumors	
*Hemangioma*	14 (0.6%)
*Cysts*	13 (0.6%)
*Focal nodular hyperplasia*	13 (0.6%)
Metastases	590 (26.5%)
Poorly differentiated carcinomas	30 (1.4%)
Echinococcosis	4 (0.2%)
Abscess	42 (1.9%)
Ascites	1 (0.04%)
Adenoma	2 (0.1%)
Bilioma	2 (0.1%)
Mucor mycosis	1 (0.04%)
Ethanol instillation (intervention)	12 (0.5%)
No pathological findings	307 (7.3%)
Non-diagnostic puncture	63 (2.8%)

*ASH* alcoholic steatohepatitis, *CCC* Cholangiocarcinoma, *HCC* Hepatocellular carcinoma, *NASH* nonalcoholic steatohepatitis, *PBC* Primary biliary cirrhosis, *PSC* Primary sclerosing cholangitis.

Complication rate was 1.2% (n = 27) with major complications in 0.5% (n = 12) and minor complications in 0.7% (n = 15). Bleeding rate was 0.9% (n = 20) with major bleeding in 0.4% (n = 8) and minor bleeding in 0.5% (n = 12). There were no puncture-related deaths. The complications are presented in Table 1.

Overall, 39.8% (n = 1064) of the punctures were performed with FNAC, 33.2% (n = 888) with CB and 26.9% (n = 719) with ABM. The respective frequencies of the documented techniques together with the respective needle diameters are presented in Table 3.

**Table 3:** Needle diameters and puncture techniques

	**Focal lesion n (%)**	**Parenchyma n (%)**
0.7 mm (FNAC)	1023 (38.3)	3 (0.1)
0.95 mm (FNAC)	38 (1.4)	0 (0)
1.2 mm (CB)	547 (20.5)	282 (10.6)
1.4 mm (ABM)	0 (0)	719 (26.9)
1.6 mm (CB)	48 (1.8)	11 (0.4)

*ABM* Aspiration biopsy (Menghini technique), *CB* Cutting biopsy, *FNAC* Fine needle aspiration cytology.

### Comparison of CB with FNAC

With respect to needle gauge, a significantly higher bleeding rate of 2.7% (4/148) was reported for biopsies of focal lesions using CB (needle diameter 1.2 mm) compared with biopsies using FNAC (needle diameter 0.7 mm) with a bleeding rate of 0.0% (0/585). Biopsies of focal lesions using both FNAC (needle diameter 0.7 mm) and CB (needle diameter 1.2 mm) also are associated with a bleeding rate of 2.2% (8/368) which is also higher that that observed for biopsies at which only FNAC (needle diameter 0.7 mm) was used (Table 4). Findings of the present study show a clear increase in bleeding complications associated with the use of thicker needles.

**Table 4:** Bleeding rate in relation to needle diameter, technique and localization (focal lesion, parenchyma)

**Localization**	**Biopsies (n)**	**Technique (Ø in mm)**	**Major BR in % (n)**	**Minor BR in % (n)**	**Total BR in % (n)**	***p***
Parenchyma	1,011	All techniques	0.4 (4)	0.3 (3)	0.7 (7)	0.3775*
Focal lesion	1,218	All techniques	0.3 (4)	0.7 (9)	1.1 (13)	
						
Parenchyma	717	ABM (1.4)	0.0 (0)	0.3 (2)	0.3 (2)	0.0255*
Parenchyma	279	CB (1.2)	0.4 (1)	1.4 (4)	1.8 (5)	
						
Parenchyma	279	CB (1.2)	0.4 (1)	1.4 (4)	1.8 (5)	0.5214*
Focal lesion	148	CB (1.2)	0.0 (0)	3.4 (5)	3.4 (5)	
						
Focal lesion	148	CB (1.2)	0.0 (0)	3.4 (5)	3.4 (5)	<0.0001**
Focal lesion	585	FNAC (0.7)	0.0 (0)	0.0 (0)	0.0 (0)	
						
Focal lesion	585	FNAC (0.7)	0.0 (0)	0.0 (0)	0.0 (0)	<0.0001**
Focal lesion	368	CB & FNAC (1.2 & 0.7)	1.1 (4)	1.1 (4)	2.7 (8)	

* Bivariate logistic regression, statistically significant values in **bold** type.

** Fisher’s exact test von Fisher, statistically significant values in **bold** type.

*ABM* Aspiration biopsy (Menghini technique), *BR* Bleeding rate, *FNAC* Fine needle aspiration cytology, *CB* Cutting biopsy.

### Comparison of CB (1.2 mm) with ABM (1.4 mm)

With respect to biopsy technique, biopsies of the hepatic parenchyma using only CB (needle diameter 1.2 mm) were associated with a bleeding rate of 1.8% (5/279). This was six times higher than the bleeding rate of 0.3% (2/717) associated with ABM (needle diameter 1.4 mm; Odds ratio [OR] = 6.52, 95%-confidence interval [CI] = [1.26-33.83], *p* = 0.03) (Table 4).

### Examiner experience

In 60.9% (n = 1,358) of biopsies, examiners were considered “inexperienced”. A complication rate of 0.7% (n = 10) was reported for these biopsies . By comparison, the 39.1% (n = 871) of biopsies performed by “experienced” examiners were associated with a 2.7-times higher complication rate of 2.0% (n = 17) at bivariate regression (OR = 2.68, 95% CI [1.22-5.89], *p* = 0.01). A total of 45 examiners performed an average 50 ± 115 liver biopsies (range 1–756 liver biopsies). While 73.3% (n = 33) of examiners caused no complications, the remaining 26.7% (n = 12) experienced between one and 14 complications (Table 5).

**Table 5:** Rate of complications in relation to the number of punctures performed by the respective examiners

**Examiner**	**Minor complication**	**Major complication**	**Total biopsies**
A	1 (1.0%)	0	96
B	1 (1.6%)	0	64
C	1 (1.9%)	0	52
D	0	1 (0.6%)	156
E	0	1 (1.3%)	78
F	0	1 (1.5%)	66
G	0	1 (1.7%)	59
H	0	1 (4.2%)	24
I	0	1 (5.6%)	18
J	1 (0.6%)	1 (0.6%)	164
K	1 (1.2%)	1 (1.2%)	86
L	7 (0.9%)	7 (0.9%)	776

### Coagulation parameters

Coagulation parameters (platelet count, Quick’s test and PTT) were fully documented for 34.0% (n = 757) of biopsies of the liver. Coagulation parameters were documented or retrospectively ascertained in 90% (18/20) of patients with bleeding complications. Bleeding rate for biopsies performed in patients meeting full coagulation criteria stood at 2.3% (16/710) compared with 4.3% (2/47) for biopsies performed in patients not fully meeting these criteria. This difference was not statistically significant on bivariate logistic regression (*p* = 0.3911). When the coagulation parameters were taken as constant variables, however, there was a decrease in bleeding rate with increasing Quick’s test values and an increase with increasing PTT values. There was no comparable correlation for platelet count (Table 6).

**Table 6:** Influence of coagulation parameters on bleeding rates

	**Platelet count**	**Quick test**	**PTT**
Reference range	≥ 70,000/μl	≥ 70 %	< 50 s
Findings in relation to biopsy	42.5% (n = 948)	43.4% (n = 968)	34.8% (n = 775)
Biopsy in patients not meeting full coagulation criteria	1.2% (n = 11)	5.1% (n = 49)	1.4% (n = 11)
Most significant deviation from reference range	32,000/μl	45%	66 s
OR [95%-CI]	1.000 [0.997-1.004]	0.970 [0.0943-0.999]	1.078 [1.014-1.146]
p-value *	0.8301	**0.0430**	**0.0157**

* Bivariate logistic regression for individual coagulation parameters considered as constant variables, statistically significant values in **bold** type.

*CI* Confidence interval, *OR* Odds Ratio, *PTT* Partial Thromboplastin Time, *s* Seconds.

### Puncture targets

Two separate analyses were performed to assess the influence of the nature of the target lesion (focal lesions vs. parenchyma) on bleeding rates: the first included all biopsies of the liver, while the second focused on those biopsies involving CB (needle diameter 1.2 mm). By excluding the factors “needle diameter” and “technique”, exact comparability could be achieved. Neither analysis found statistically significant differences with respect to bleeding rate for the different puncture targets (*p* = 0.35; *p* = 0.30; Table 2).

### Diameter of lesions

Diameter of the target lesion was documented for 1,090 biopsies. Broken down according to size, 18.0% (n = 196) of biopsies were performed in patients with target lesions < 2 cm in diameter with a bleeding rate of 1.0% (n = 2); 56.2% (n = 613) with lesions 2–5 cm in diameter with a bleeding rate of 1.3% (n = 8); and 25.8% (n = 281) with lesions > 5 cm in diameter with a bleeding rate of 1.1% (n = 3). With respect to bleeding rate, no statistically significant difference between the three size categories could be ascertained using Fisher’s exact test (*p* = 1.0).

### Sex and age

Similarly, bivariate logistic regression failed to demonstrate any difference for complication rate of 0.8% (n = 10) among 1,238 biopsies in male patients vs. 1.7% (n = 17) among 991 biopsies in female patients (*p* = 0.06). In 82.6% of liver biopsies (n = 1842), punctures were performed in patients < 70 years of age, with a bleeding rate of 0.7% (n = 13). In the remaining 17.4% (n = 387) of biopsies performed in patient ≥ 70 years of age, the bleeding rate was 1.8% (n = 7) representing an increase of 2.6 times at bivariate logistic regression (OR = 2.60; 95%-CI [1.03-6.54]; *p* = 0.04).

## Discussion

A comparison of complication rates with other studies can be done most objectively when consideration is limited to complications involving bleeding. Our bleeding rates of 0.4% for major bleeding and 0.5% for minor bleeding fall toward the lower end of the ranges reported in the literature for both major (0.0-5.3%) and minor (0.0-5.9%) bleeding [2,7]. The rates for minor bleeding in particular are difficult to interpret since these complications may be detected at ultrasonographic monitoring examinations but may be otherwise clinically inapparent. Studies suggest that minor intra- or perihepatic bleeding detectable by ultrasound occurs in about 18-20% of liver biopsies [2,11,12]. Ultrasonographic monitoring is, however, not routine in all centers performing liver punctures. In our department, it is routine following CB and ABM but not following FNAC. Our mortality rate of 0.0% corresponds with the very low rate of puncture-related mortality of 0.0-0.4% documented in the literature [2,13-15].

With respect to needle gauge, our findings correspond with data reported from two studies using anesthetized pigs in which bleeding rate correlated with increasing diameter of the puncture needles [16-18]. In humans, a similar correlation was observed in only one large prospective study [19]. In that study, needles with diameters of 1.8 mm and 1.6 mm were associated with a significantly higher complication rate than were thinner needles 1.2 mm and 0.8 mm in diameter. Analysis of the data, however, included findings from punctures of several different organs; in addition, computed tomography served as the imaging method and the overall complication rate, rather than simply assessment of bleeding complications, was studied. This significantly limits a direct comparison with the findings of the present study. By contrast, two other studies failed to observe an increased rate of bleeding complications in liver punctures using larger-gauge needles [20-22]. Because of the available data, however, these results do not appear to justify any corresponding conclusions. In one study, there is a lack of exact information on the number of punctures performed with each respective needle diameter [21]; in the other, the number of cases is too small to allow valid conclusions regarding the otherwise very low bleeding rate [20]. In fact, the currently available data do not satisfactorily demonstrate the independence of bleeding rate from needle diameter. On the other hand, because of the large number of cases, clearly described needle diameters and the exclusive analysis of data obtained from biopsies of focal lesions, we consider our findings to be very reliable and to clearly demonstrate a significantly lower bleeding rate with the use of thinner needles. The impact of different puncture techniques (FNAC vs. CB) on these results is, in our opinion, negligible. Both were performed in nearly identical manner with respect to both the ultrasonographic guidance of the liver biopsies and the duration of the intrahepatic phase (5–30 seconds).

In the case of punctures of the hepatic parenchyma, bleeding rate associated with CB (needle diameter 1.2 mm) was approximately six-times higher than that reported for ABM (needle diameter 1.4 mm). This correlation has not previously been adequately explained although this had been suggested by data reported for early retrospective studies [2,9,23,24].

An important consideration in the comparison of these two methods is the fact that, unlike ultrasonographically guided CB, ABM is performed with ultrasonographic support but not under real time conditions. However, real-time imaging does not appear to reduce the bleeding risk: significant bleeding usually results from arterial sources but small arteries cannot be visualized at ultrasound. By contrast, the constant ultrasonographic monitoring does reduce the risk of injury to the lung and gallbladder [2,25]. The reason for the reduced bleeding rate with ABM, in our opinion, is the shorter intrahepatic phase of the puncture needle (ca. 1–3 seconds). Respiration-associated injuries such as tears of the liver capsule are less likely for this reason than with CB punctures, in which the intrahepatic phase in our institution lasts 5–30 seconds depending on puncture circumstances. This explanation has also been proposed by Grant et al. [3]. For the indication of histological assessment of cirrhosis, however, CB appears to be superior to ABM [2,26,27].

With respect to examiner experience, a direct comparison between studies is difficult due to differing definitions regarding the respective examiners’ degree of experience. Only the multi-center study reported by Cadranel et al. [28] used the same breakdown of examiner experience selected for the present study. That study, however, showed a significant reduction in complication rate, both for minor complications, such as pain and feeling unwell, and for major complications in relation to increasing examiner experience. Other publications use different criteria for determining examiner experience but report similar findings [18,21,29-31]. In the study by Cadranel et al., examiners with more than 150 puncture procedures were considered experienced, while those with less than 15 procedures were considered inexperienced. No significant difference in complication rates was reported between these two groups [28].

In our collective, however, complication rate increase in relation to examiner experience and do so in a statistically significant manner. This unexpected observation can be explained by the fact that difficult, “high risk” biopsies are only performed by experienced examiners; the elevated complication rate can thus be attributed to the more difficult puncture conditions. Inexperienced examiners are initially assigned patients with secure puncture conditions and perform procedures under supervision. In our opinion, the clear assignment of each biopsy to the corresponding degree of examiner experience serves to underscore our findings and could be interpreted to indirectly support the findings of other authors that examiner inexperience is not a risk factor for increased complication rates [10,18,21]. This also underscores the importance of careful supervision and a gradual advancement of less experienced examiners to increasingly complex puncture situations. It should be noted, that the results were exclusively calculated bivariate and therefore other factors are not taken into account.

Our data support the conclusion of other publications that normal or only mildly reduced coagulation parameters do not prevent bleeding complications [2,3,7,9,10,21,22]. Overall, 88.9% (16/18) of instances of bleeding in the present study in patients with documented coagulation parameters occurred in patients whose coagulation parameters were within the normal reference range. Thus, our pre-interventional coagulation screening would not seem to have protected against bleeding events. Reports in the literature have found thromboplastin and PTT to be poor predictors of bleeding risk in association with surgical procedures [1], however, based on our findings, we do not believe that there is justification for dispensing with pre-interventional screening. By comparison, Steeff et al. found a significant correlation between the occurrence of a complication and a platelet count < 60,000/mm^3^; hence, these researchers advised against puncture biopsies in patients with low platelet counts [8]. Patients with end-stage liver diseases may experience more significant bleeding [32,33]. Considering decreasing PTT and increasing thromboplastin as constant variable, we were able to ascertain a decrease in bleeding complications. A similar correlation for the platelet count did not emerge in the statistical treatment of the data.

A comparison of punctures of focal lesions vs. parenchymal punctures failed to document an increase bleeding rate. This supports data published by Frieser et al. in the only study available to us that addresses this issue [4]. The absence of correlation between complication rate and lesion size confirms findings by Ch Yu et al. [34], who, in his study of puncture-related bleeding rate in patients with hepatocellular carcinoma (HCC), established four size categories, finding no statistically significant differences in terms of complication rates.

In comparison with other studies [10,28], our data did not support any sex-specific elevation in risk. Cadranel et al. [25] report that “pain” and “fear” assessed using a 10-point visual analog scale occurred more frequently in female patients. In our opinion, these parameters, because of their subjectivity, do not seem useful for a gender comparison. On the other hand, McGill et al. [10] reported an increased risk of bleeding rate in relation to the factor “sex” in combination with age, underlying malignancy and increase number of needle passes that was found to be statistically significant at multivariate logistic regression. Patients in our study aged 70 years and older showed a clearly higher rate of bleeding complications than did younger patients. An age-independent complication rate, as has been reported in a large study by Welch et al. [35], could not be confirmed by our data. In Welch’s study, the complication rate for patients > 80 years of age was compared with that observed for younger patients. However, analysis of the data considered all complications, not simply bleeding: hence, a direct comparison is difficult. When bleeding rates alone are considered, our findings confirm data reported by McGill et al. [29], which, on bivariate logistic regression, showed significantly more frequent bleeding in relation to increase patient age.

## Conclusions

In conclusion, the findings of the present study show elevated rates of bleeding when larger-caliber puncture needles are used. A comparison of CB with ABM also showed an increased risk for CB. A lesser degree of examiner experience, on the other hand, did not represent a risk factor for elevated complication rates. The coagulation parameters thromboplastin and PTT significantly affect the bleeding rate. A similar influence was not observed for platelet count. For the factors puncture target (focal lesion vs. parenchyma), patient sex and lesion side, for all of which there is a general paucity of available data, our findings did not demonstrate any significant differences with respect to bleeding rate or complication rate. Advanced age, however, was clearly associated with an increased rate of bleeding complications.

## Abbreviations

AASLD: American Association for the Study of Liver Diseases; ABM: Aspiration biopsy (Menghini technique); CB: Cutting biopsy; CI: Confidence interval; FNAC: Fine needle aspiration cytology; OR: Odds ratio; PTT: Partial thromboplastin time.

## Competing interests

The authors declare that they have no competing interests.

## Authors’ contribution

MM: study design, data collection, article preparation, article writing; WK: conception of the study, study design, data collection, article preparation, article writing, article review; SO: article preparation, article writing, article review; MW: data analysis, article preparation; RM: article preparation, article review; RAM: article preparation, article writing; MMH: study design, data collection, article preparation, article review. All authors read and approved the final manuscript.

## Pre-publication history

The pre-publication history for this paper can be accessed here:

http://www.biomedcentral.com/1471-230X/12/173/prepub
